# Facile and ecofriendly green synthesis of Co_3_O_4_/MgO–SiO_2_ composites towards efficient asymmetric supercapacitor and oxygen evolution reaction applications†

**DOI:** 10.1039/d4ra07337a

**Published:** 2024-11-28

**Authors:** Gulzar Ali, Aneela Tahira, Asma Hayat, Muhammad Ali Bhatti, Aqeel Ahmed Shah, Syed Nizam Uddin Shah Bukhari, Elmuez Dawi, Ayman Nafady, Riyadh H. Alshammari, Matteo Tonezzer, Muhammad Kashif Samoon, Zafar Hussain Ibupoto

**Affiliations:** a Institute of Chemistry, University of Sindh Jamshoro 76080 Pakistan gulzaralichemist@gmail.com asmabaloch141617@gmail.com zaffar.ibhupoto@usindh.edu.pk; b Institute of Chemistry, Shah Abdul Latif University Khairpur Mirs Sindh Pakistan aneela.tahira@salu.edu.pk; c Centre for Environmental Sciences, University of Sindh Jamshoro 76080 Sindh Pakistan mali.bhatti@usindh.edu.pk; d Wet Chemistry Laboratory, Department of Metallurgical Engineering, NED University of Engineering and Technology, University Road Karachi 75270 Pakistan aqeelshah@cloud.neduet.edu.pk; e Department of Basic Science and Humanities, Dawood University of Engineering and Technology Karachi Sindh 74800 Pakistan nizamuddin@duet.edu.pk; f College of Humanities and Sciences, Department of Mathematics and Sciences, Ajman University P. O. Box 346 Ajman UAE e.dawi@ajman.ac.ae; g Chemistry Department, College of Science, King Saud University Riyadh 11451 Saudi Arabia anafady@ksu.edu.sa ralshammari@ksu.edu.sa; h Department of Chemical and Geological Sciences, University of Cagliari Monserrato Italy matteo.tonezzer@cnr.it; i Centre for Pure and Applied Geology, University of Sindh Jamshoro Jamshoro Sindh 76080 Pakistan kashif.samoon@usindh.edu.pk

## Abstract

The development of low-cost, eco-friendly, and earth-friendly electrode materials for energy storage and conversion applications is a highly desirable but challenging task for strengthening the existing renewable energy systems. As part of this study, orange peel extract was utilized to synthesize a magnesium oxide-silicon dioxide hybrid substrate system (MgO–SiO_2_) for coating cobalt oxide nanostructures (Co_3_O_4_) *via* hydrothermal methods. A variety of MgO–SiO_2_ compositions were used to produce Co_3_O_4_ nanostructures. The purpose of using MgO–SiO_2_ substrates was to increase the porosity of the final hybrid material and enhance its compatibility with the electrode material. This study investigated the morphology, chemical composition, optical properties, and functional group properties. In hybrid materials, the shape structure is inherited from nanoparticles with uniform size distributions that are well compacted. A relative decrease in the optical band was observed for Co_3_O_4_ when deposited onto an MgO–SiO_2_ substrate. An improvement in the electrochemical properties of Co_3_O_4_/MgO–SiO_2_ composites was observed during the measurements of supercapacitors and oxygen evolution reaction (OER) in alkaline solutions. The Co_3_O_4_/MgO–SiO_2_ composite prepared on 0.4 g of the MgO–SiO_2_ substrate (sample 2) demonstrated excellent specific capacitance, high energy density, and recycling stability for 40 000 galvanic charge–discharge cycles. The assembled asymmetric supercapacitor (ASC) device demonstrated a specific capacitance of 243.94 F g^−1^ at a current density of 2 A g^−1^. Co_3_O_4_/MgO–SiO_2_ composites were found to be highly active towards the OER in 1 M KOH aqueous solution with an overpotential of 340 mV at 10 mA cm^−2^ and a Tafel slope of 88 mV dc^−1^. It was found that the stability and durability were highly satisfactory. Based on the use of orange peel extract, a roadmap was developed for the synthesis of porous hybrid substrates for the development of efficient electrode materials for energy storage and conversion.

## Abstract 2

The development of low-cost, eco-friendly, and earth-friendly electrode materials for energy storage and conversion applications is a highly desirable but challenging task for strengthening the existing renewable energy systems.

## Graphical abstract

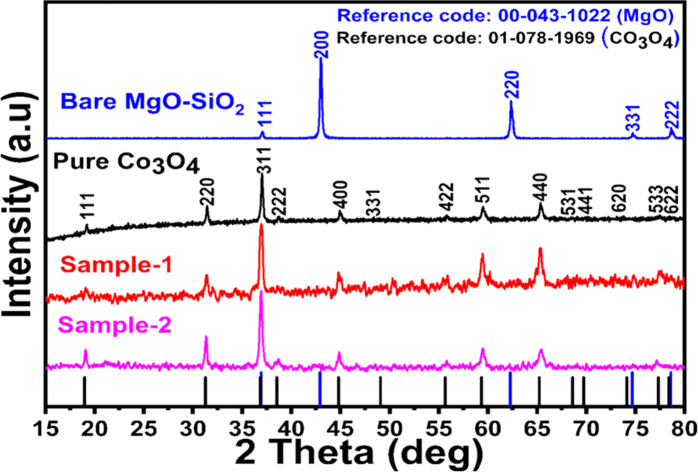

## 1. Introduction

Among the most important aspects of energy storage devices is the use of non-polluting and extremely stable energy materials. Over the past few decades, the demand for renewable energy sources and storage systems has increased significantly owing to the widespread use of non-renewable energy sources. In response, significant pollution rise has been observed globally.^1,2^ It is of the utmost importance that novel research activities be undertaken to develop renewable energy sources and energy storage devices to meet the demand and eliminate the possibility of pollution. In recent years, batteries have been widely used to store direct renewable energy derived from a variety of renewable sources; however, their power density is underrated. The benefits of supercapacitors include rapid charging times, long cycling stability, safety, excellent power density, and low development costs.^3–6^ Supercapacitors are therefore considered an attractive energy storage system for resolving the storage issue associated with renewable energy sources. Based on their charge-storing mechanisms, it has been determined that supercapacitors can be divided into two types. Electric double layer capacitors (EDLCs) are based on an ion adsorption principle, and they mostly use carbon-based materials, except for ruthenium oxide (RuO_2_). Another type of capacitor is a pseudocapacitor, which works on the principle of storing charge through Faradaic mechanisms. Pseudo-capacitors, such as bimetallic nickel–cobalt oxide, are common mechanisms by which metal oxides and chalcogenides store charge. In evaluating the performance of supercapacitors, it is important to consider the efficiency of charge storing. For the development of supercapacitors, conducting polymers, metal chalcogenides, and metal hydroxides, such as carbon nanotubes and graphene oxide, metal oxides, conducting polymers, and metal hydroxides have been used. The high stability and tunable oxidation states of transition metal oxides make them excellent materials for supercapacitors. Cobalt oxide (Co_3_O_4_) has been widely employed in the development of supercapacitors due to its enhanced electrochemical activity, excellent reversibility, and high theoretical capacity of 3560 F g^−1^.^7–9^ Several methods have been described for the preparation of Co_3_O_4_ and its composite systems for use in energy storage, including electrodeposition, chemical baths, co-precipitation, spray pyrolysis, sol–gel, electrospinning, *etc.* A variety of shapes of Co_3_O_4_ have been obtained, including nanorods and flowers, and their size and morphology have been evaluated as a function of supercapacitor performance. To enhance cycling stability and charge transport, silicon dioxide is incorporated into the electrode material.^10^ Furthermore, magnesium oxide (MgO) is a relatively abundant, cost-effective, environmentally friendly, and significant catalyst for the creation of composite systems for water separation.^11,12^ There is evidence that MgO enhances the catalytic activity of composite materials containing a small amount of the substance.^13^ The use of MgO and SiO_2_ together towards electrode performance has several merits such as MgO offering enhanced cycling stability and lifetime of the electrode material.^14^ Besides this, MgO is low cost, ecofriendly and offers large scale production. SiO_2_ is low cost, environmentally friendly and highly utilized as a raw material in the fabrication of active anode materials for enhancing the charge storage capacitance.^15^ Enhanced energy storage performance could be obtained from an efficient hybrid material *via* unique structural properties of SiO_2_. Hence, the development of hetero configurations of electrode materials having a modified surface enables multiple advantages such as frequent access of electrolytic ions for rapid redox processes and, consequently, improved electrochemical activity can be achieved. As a result of its high energy density and non-emission of carbon dioxide into the atmosphere, there should be a great deal of emphasis placed on implementing intensive measures to produce green hydrogen.^16^ Water electrolysis is the most suitable method for generating hydrogen for industrial, transportation, and domestic purposes.^17^ The splitting of water occurs as a result of the oxygen and hydrogen evolution reactions (OER, HER). However, electrochemical water splitting is limited by large overpotentials due to the disparity between experimental and thermodynamic electrochemical potentials. Water electrolysis, or HER, relies on a two-electron transfer mechanism. OER, on the other hand, involves a four-electron electron transfer mechanism,^18,19^ indicating that OER is a relatively more complicated and energy-intensive process requiring an efficient electrocatalyst.^20^ These aspects highlight the global challenges in the shortage of non-renewable energy resources, producing easily accessible renewable energy sources, and strengthening the electrochemical energy related devices. Further, it is highly necessary to fabricate electrode materials for electrochemical energy conversion and storage devices using low-cost, nonprecious metals and ecofriendly characteristics. For these reasons, our aim was to investigate composite materials based on Co_3_O_4_ towards energy conversion and energy storage devices. This could enable the strengthening of hydrogen production *via* electrochemical water splitting as an energy conversion application. And at the same time, we studied the capacitance of these electrode materials for the development of energy storage devices. This study is the first time reporting Co_3_O_4_/MgO–SiO_2_ composites developed by using a modified hydrothermal process with orange peel extract. Co_3_O_4_/MgO–SiO_2_ composite exhibits excellent electrochemical performance in alkaline electrolytes owing to its abundant catalytic sites, high electrode compatibility, and rapid charge transfer. As a first step, the MgO–SiO_2_ composite was grown *in situ* using orange peel extract, followed by coating Co_3_O_4_ nanostructures on top of MgO–SiO_2_.

## 2. Experimental section

### 2.1. Chemical reagents

Cobalt chloride hexahydrate (98%), aqueous ammonia solution (33%), potassium hydroxide (99%), acetone (99.9%), ethanol (99.5%), urea (99%) and 5% Nafion were purchased from Sigma-Aldrich (Sindh Karachi, Pakistan) and used as received. The chemical reagent solution was made in deionized water. The orange fruit was received from a local Jamshoro Sindh, Pakistan, market.

### 2.2. Synthesis of Co_3_O_4_/MgO–SiO_2_ composites using modified hydrothermal method

To begin with, orange peels were washed with deionized water. After drying, they were ground into small pieces and converted into powder. One liter of deionized water was added to 250 grams of orange peel, and the mixture was continuously magnetically stirred for 24 hours. The orange peel extract was obtained by filtration after 24 hours. In addition, 200 mL of orange extract was treated with 0.5 M silica and magnesium nitrate hexahydrate. Aqueous ammonium hydroxide solution was added to the solution in order to maintain the pH at 9.6. Following this, the growth solution was placed in an oven at 80 °C for two hours. A filter was placed over the sample and it was allowed to dry at room temperature for two hours. Afterwards, the grown sample was placed in a crucible and placed in a muffle furnace for four hours at 600 °C. A white product consisting of bare MgO and SiO_2_ was received next. Finally, the different masses of bare MgO–SiO_2_ composite were placed into two beakers labeled sample 1 and sample 2, and then 0.1 M cobalt chloride hexahydrate and 0.1 M urea were added with a volume of 100 mL to each beaker. Once the growth solution had been homogenized, both beakers were covered with an aluminum sheet and placed in an electric oven at 95 °C for 5 hours. Before the growth process, the pH of growth solutions of sample 1 and sample 2 were found to be 4.95 and 4.6, and after the growth process they slightly decreased to 4.84 and 4.43, respectively. The pure Co_3_O_4_ was prepared by dissolving 0.1 M cobalt chloride hexahydrate and 0.1 M urea in a volume of 100 mL with a pH of 5.25. The pH of the pure Co_3_O_4_ solution after the growth process was about 5.1. After the growth process, the product was filtered and dried at room temperature. Later, thermal annealing was carried out at 500 °C for 5 hours. The crystal phase and purity of the synthesized material were investigated using a PAnalytic X'Pert PRO X-ray diffractometer using measurement conditions of 10 to 80 two theta, 0.02 step, and 0.3 seconds. The morphology was studied by employing a Carl Zeiss EVO-18 instrument using an accelerating voltage of 10 kV. The optical band gap studies were done using a UV-visible spectrophotometer *via* absorbance spectra in the 200–700 nm range on a Shimadzu UV-1900 UV-visible spectrophotometer. Functional group studies were obtained through the FTIR spectrum in the 4000–400 cm^−1^ range on a Thermo Nicolet 380 FTIR instrument with the KBR pellet technique.

### 2.3. Electrochemical studies of Co_3_O_4_/MgO–SiO_2_ composites

All electrochemical experiments were conducted on a Versa potentiostat. In order to conduct preliminary studies on supercapacitors and OERs, a three-electrode cell set-up was used. The synthesized materials served as working electrodes, platinum wire as counter, and silver–silver chloride (Ag/AgCl) filled with 3 M KCl solution. Glassy carbon electrodes (GCEs) were used as current collectors by depositing the catalytic inks of the presented materials. The GCE was cleaned with 0.03 μm alumina paste, rubbed with silicon paper, and washed with ethanol and water. The synthesized materials were mixed with 3 mL of deionized water and 0.5 mL of 5% Nafion to produce catalytic ink. A mass (0.02 mg) of catalytic ink of 5 L was deposited on the GCE and dried using the blower for the OER activity analysis. The current collector electrode in the supercapacitor was nickel foam due to its large surface area and ability to hold more electrode material after mild sintering at 70 °C for 30 minutes. The catalytic material was deposited on nickel foam through several dip coatings, and the mass deposited was approximately 0.2 mg. For the purposes of the OER studies, 1 M KOH electrolytic solution was used. The supercapacitor measurements were made in 3 M KOH due to greater adsorption of electrolytic ions for improved electrode performance. For the electrochemical performance evaluation of the synthesized materials, several electrochemical methods were used, including cyclic voltammetry (CV), linear sweep voltammetry (LSV), chronoamperometry, chronopotentiometry, and electrochemical impedance spectroscopy (EIS). The EIS study was conducted using a frequency range of 100 kHz to 0.1 Hz, an amplitude of 5 mV, and an onset potential of 0.1 Hz.

## 3. Results and discussion

### 3.1. Various structural studies of Co_3_O_4_/MgO–SiO_2_ composite

**Fig. 1:**
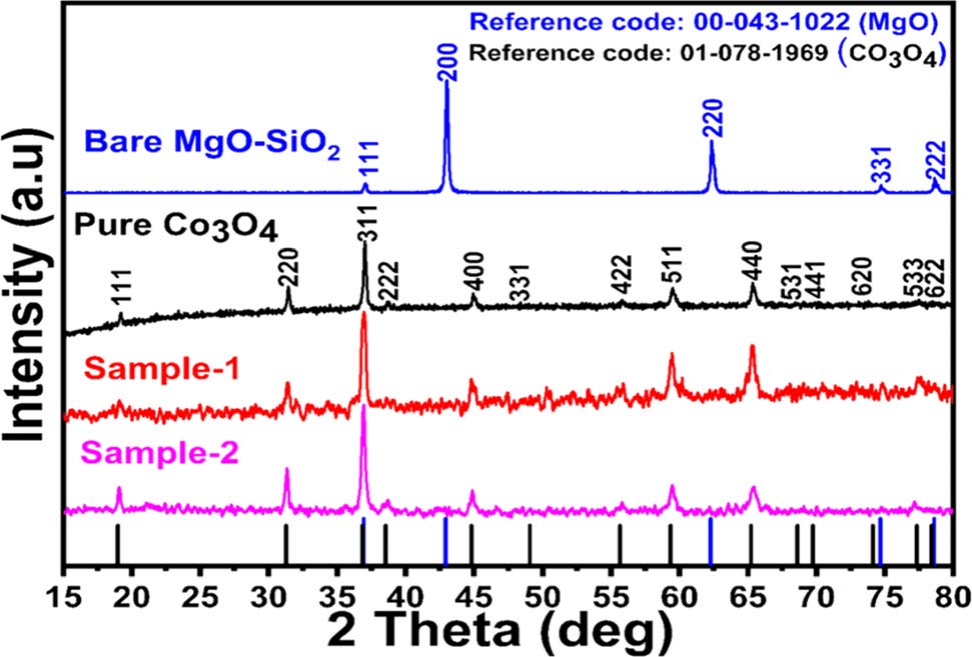
XRD reflection peaks of pure Co_3_O_4_, bare MgO–SiO_2_ and Co_3_O_4_/MgO–SiO_2_ composites (sample 1 and sample 2).

As shown in Fig. 1, XRD was used to identify the crystal phase of the Co_3_O_4_ system, and the measured diffraction pattern indicated that the samples mainly contained Co_3_O_4_ without any impurities. A typical cubic phase was also observed in the Co_3_O_4_/MgO–SiO_2_ composite with MgO diffraction patterns. However, the effect of different MgO–SiO_2_ compositions on the diffraction patterns of Co_3_O_4_/MgO–SiO_2_ composites can be observed through the relative intensities of the diffraction patterns of each composite sample. Due to its potentially amorphous nature, SiO_2_ was not detected in silica gel. According to the XRD results, the material's crystal properties have been significantly altered, which may have allowed it to expose its favorable crystal planes for electrochemical reactions.

**Fig. 2:**
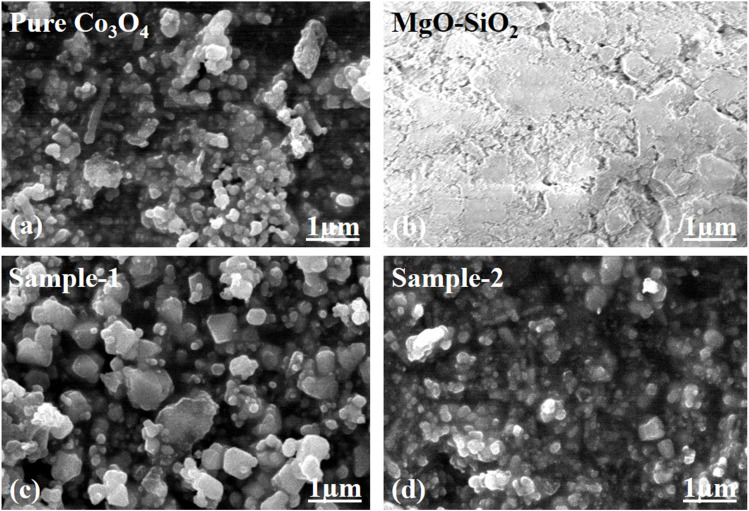
Distinctive SEM images of (a) pure Co_3_O_4_, (b) bare MgO–SiO_2,_ (c) Co_3_O_4_/MgO–SiO_2_ composite (sample 1), and (d) Co_3_O_4_/MgO–SiO_2_ composite (sample 2).

**Fig. 3:**
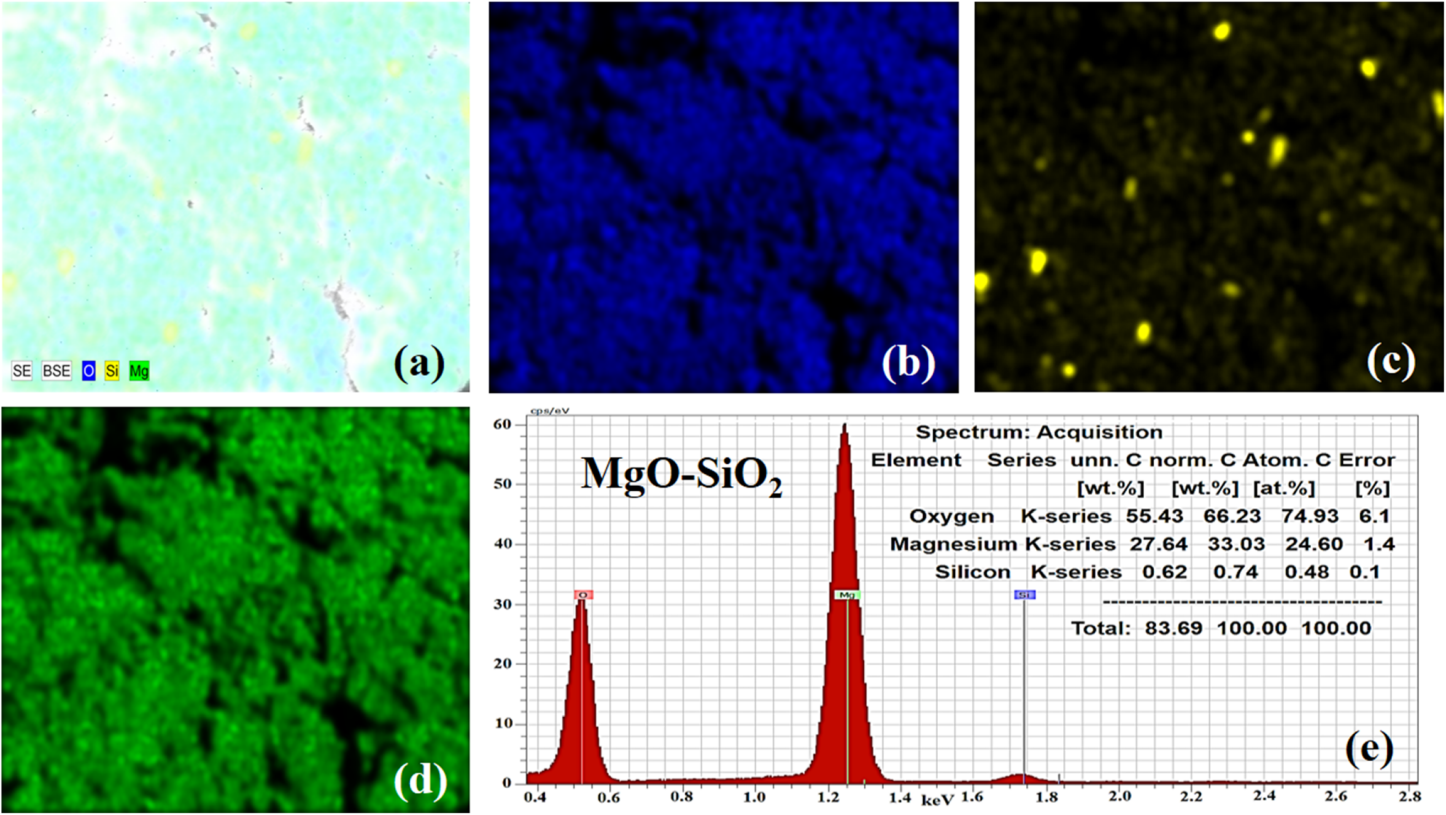
(a) Elemental mapping of MgO–SiO_2_. (b–d) Elemental mapping of Mg, Si, and O. (e) EDX spectrum of the bare MgO–SiO_2_ composite.

Using SEM analysis, we studied the shape structure of a pure Co_3_O_4_ system, bare MgO–SiO_2_ composite, and two samples of Co_3_O_4_/MgO–SiO_2_ composite (sample 1, sample 2). SEM analysis of the Co_3_O_4_ system revealed irregular nanoparticles with heterogeneous morphologies, as shown in Fig. 2a. As shown in Fig. 2b, the SEM micrograph shows the bare MgO–SiO_2_ composite oriented with a typical cluster morphology and dimensions of several hundreds of nanometers. The SEM images of the Co_3_O_4_/MgO–SiO_2_ composites (sample 1 and sample 2) were also analyzed for morphological aspects, as shown in Fig. 2c and d. As shown in Fig. 3d, adding more MgO–SiO_2_ composite during the growth of Co_3_O_4_ (sample 2) resulted in well-packed nanoparticles with uniform distribution and dimensions less than 200 nm. However, sample 1 of the Co_3_O_4_/MgO–SiO_2_ composite had slightly larger nanoparticles with weak packing and 200–300 nm dimensions, as shown in Fig. 2c. SEM analysis revealed that sample 2 of the Co_3_O_4_/MgO–SiO_2_ composite has relatively smaller particle sizes and a high structural compatibility, enabling better adhesion with the electrode surface for improved electrochemical activity. The decrease in the size of Co_3_O_4_/MgO–SiO_2_ composite (sample 2) could be attributed to the slight variation in pH towards acidic pH during the growth process, which might cause an etching effect.

**Fig. 4:**
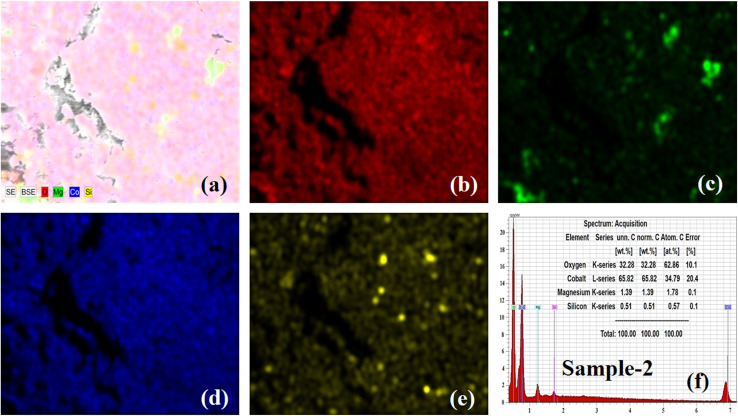
(a) Elemental mapping of the Co_3_O_4_/MgO–SiO_2_ composite (sample 2). (b–e) Elemental mapping of O, Mg, Co, and Si of the Co_3_O_4_/MgO–SiO_2_ composite (sample 2). (f) EDX spectrum of the Co_3_O_4_/MgO–SiO_2_ composite (sample 2).

Furthermore, elemental mapping and energy dispersive X-rays (EDX) analysis were done on the bare MgO–SiO_2_ composite and Co_3_O_4_/MgO–SiO_2_ composite (sample 2). The elemental mapping and EDX of the bare MgO–SiO_2_ composite are shown in Fig. 3a–e. The typical combined map of Mg, O and Si is shown in Fig. 3a. It could be seen that the sample was homogenous with the distribution of Mg, O and Si elements, as shown in Fig. 3c and d, and they were also found in the EDX spectrum, as shown in Fig. 3e. Elemental mapping and EDX were also done on the Co_3_O_4_/MgO–SiO_2_ composite (sample 2), and the corresponding data are shown in Fig. 4a–f. The combined mapping image is shown in Fig. 4a, and the uniform distribution of Co, O, Mg, and Si is shown in Fig. 4b–e. The EDX spectrum shows significant atomic % of Co, O, Mg and Si for the verification of the elemental composition of sample 2.

**Fig. 5:**
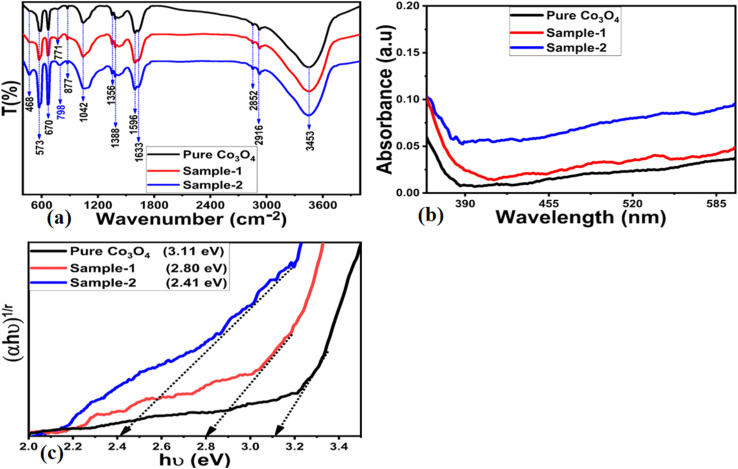
Distinctive FTIR spectra of (a) pure Co_3_O_4_ and Co_3_O_4_/MgO–SiO_2_ composites (sample 1 and sample 2), (b) UV-visible spectra of pure Co_3_O_4_ and Co_3_O_4_/MgO–SiO_2_ composites (sample 1 and sample 2), and (c) corresponding Tauc plots of pure Co_3_O_4_ and Co_3_O_4_/MgO–SiO_2_ composites (sample 1 and sample 2).

FTIR measurements were conducted on various materials, such as bare MgO–SiO_2_ composites, Co_3_O_4_, and Co_3_O_4_/MgO–SiO_2_ composites (sample 1 and sample 2). Fig. 5a depicts the corresponding IR spectra. In all materials, the IR peaks at 3453 cm^−1^ and 573 cm^−1^ corresponded to the O–H and Co–O bonds, respectively, as shown in Fig. 5a. Additionally, the clear peak at 3453 cm^−1^ in the Co_3_O_4_/MgO–SiO_2_ composites (samples 1 and 2 in the case of MgO–SiO_2_) may be due to the stretching vibration of the O–H bond, which may be from water adsorbed on the O–H bond or hydrated silica.^21^ In contrast, the IR bands at 2852–2916 cm^−1^ can be attributed to stretching vibrations of saturated C–H bonds. The IR band at 1042 cm^−1^ could be attributed to the Si–O–Si bond, and the IR vibration at 573 cm^−1^ to the Si–O bond.^22^ IR bands located at 877 cm^−1^ may be associated with Si–O stretching modes, and IR bands at 468 cm^−1^ may be associated with Mg–O stretching or Si–O bending modes. The IRT bands at 670 and 468 cm^−1^ have previously been attributed to Mg–O.^23^ According to the FTIR analysis, the composite systems contain IR bands for Mg–O, Si–O, and Co–O, indicating the presence of Co_3_O_4_, MgO, and SiO_2_.

Optical band gap studies were conducted on Co_3_O_4_ and Co_3_O_4_/MgO–SiO_2_ composites (sample 1 and sample 2) to verify the effects of adding different amounts of MgO–SiO_2_ composites during the synthesis Co_3_O_4_/MgO–SiO_2_ composites (samples 1 and 2). Fig. 5b shows the absorbance spectra of Co_3_O_4_ and Co_3_O_4_/MgO–SiO_2_ composites (samples 1 and 2). As can be seen, each spectrum is associated with two discrete absorption edges that begin at 300–320 nm and 415–450 nm. A mix of oxidation states may be responsible for these absorption edges.^24–27^ As shown in Fig. 5c, Tauc plots were used to determine the optical band gaps for Co_3_O_4_ and Co_3_O_4_/MgO–SiO_2_ composites (sample 1 and sample 2), with corresponding values of 3.11, 2.80, and 2.41 eV, respectively. According to the optical band gap analyses of the composite systems, particle size, shape, and impurities significantly influence the band gap variation of the material to be synthesized. The optical band gap analysis revealed that sample 2 had a low band gap value, which may have accelerated the charge transfer process during the electrochemical reaction.

### 3.2. Electrochemical characterization of as-synthesized Co_3_O_4_/MgO–SiO_2_ composites

**Fig. 6:**
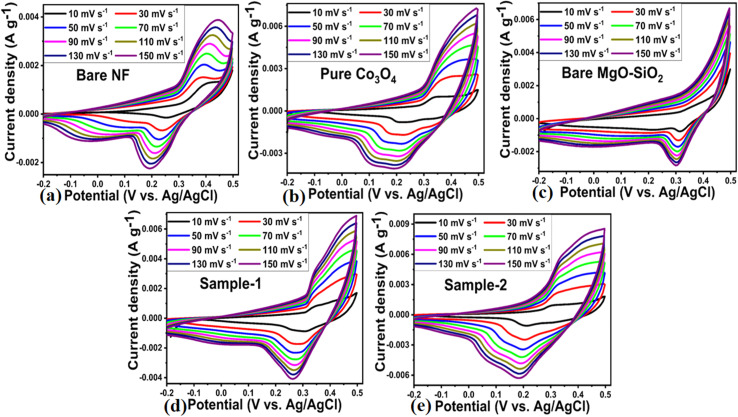
CV polarization curves at different sweeping rates in 3 M KOH aqueous solution of (a) bare nickel foam, (b) pure Co_3_O_4_, (c) bare MgO–SiO_2,_ (d) Co_3_O_4_/MgO–SiO_2_ composite (sample 1), (e) Co_3_O_4_/MgO–SiO_2_ composite (sample 2).

Based on the SEM study, a composite electrode composed of Co_3_O_4_/MgO–SiO_2_ (sample 2) was found to enhance the electrode's compatibility with the electrolyte and expose a large surface area to the electrolyte, resulting in significant improvement in and reduction of diffusion channels during charging and discharge measurements. Because Co_3_O_4_ is a transition metal oxide, it offers the highest theoretical storage capacity for electrochemical energy storage systems. At the same time, the MgO–SiO_2_ composite enhances charge transport at the interface, compatibility with electrode surfaces, and surface area for electrolytic ions. Combining Co_3_O_4_ with MgO–SiO_2_ composite shows an excellent electrode configuration for enhancing electrochemical energy storage. As shown in Fig. 6a–e, CV curves were measured for bare nickel foam, pure Co_3_O_4_, bare MgO–SiO_2_ composite and Co_3_O_4_/MgO–SiO_2_ composites (samples 1 and 2) at various scanning rates in 3 M KOH aqueous solution.

The CV curves at varying scan rates were characterized by anodic and cathodic peaks that describe the distinctive Faradaic reactions for the developed pseudocapacitors. Based on the composition of the Co_3_O_4_/MgO–SiO_2_ composites, the proposed electrodes exhibit charging and discharging processes as described in the following equations.^28,29^

1 Co_3_O_4_ + OH^−^ + H_2_O ↔ 3CoOOH + e^−^

The CV curves measured for the bare nickel foam, pure Co_3_O_4_, bare MgO–SiO_2_, Co_3_O_4_/MgO–SiO_2_ composite (sample 1), and Co_3_O_4_/MgO–SiO_2_ composite (sample 2) at various scan rates indicated a pair of redox peaks, displaying charge storage mechanisms *via* pseudocapacitive activity.^29^ The redox peak originated from reversible redox processes. All the materials, through CV curves, showed the same curves at different current densities, and there was linear increase in the area under the curves with increasing scan rates. The surface microstructure and elemental composition of the active electrode material might contribute to the large area under the CV curve. The CV curves exhibited peak potential shifts for the anodic peaks towards positive potential and for the cathodic peaks towards negative potential with increasing scan rate from 10 mV s^−1^ to 150 mV s^−1^ due to the polarization of the active electrode materials at higher sweeping scan rates.^28^ Measured shifts in anodic peak potential from 10 mV s^−1^ to 150 mV s^−1^ for the bare nickel foam, pure Co_3_O_4_, bare MgO–SiO_2,_ Co_3_O_4_/MgO–SiO_2_ composite (sample 1), and Co_3_O_4_/MgO–SiO_2_ composite (sample 2) from 0.368 to 0.444, 0.344 to 0.428, 0.359 to 0.368, 0.329 to 0.348, and 0.3042 to 0.344 V *vs.* Ag/AgCl, respectively, were observed. Also, cathodic peak potential shifts were noticed from 0.249 to 0.192, 0.315 to 0.301, 0.215 to 0.159, 0.302 to 0.263, and 0.210 to 0.184 V *vs.* Ag/AgCl for bare nickel foam, pure Co_3_O_4_, bare MgO–SiO_2,_ Co_3_O_4_/MgO–SiO_2_ composite (sample 1), and Co_3_O_4_/MgO–SiO_2_ composite (sample 2), respectively.

**Fig. 7:**
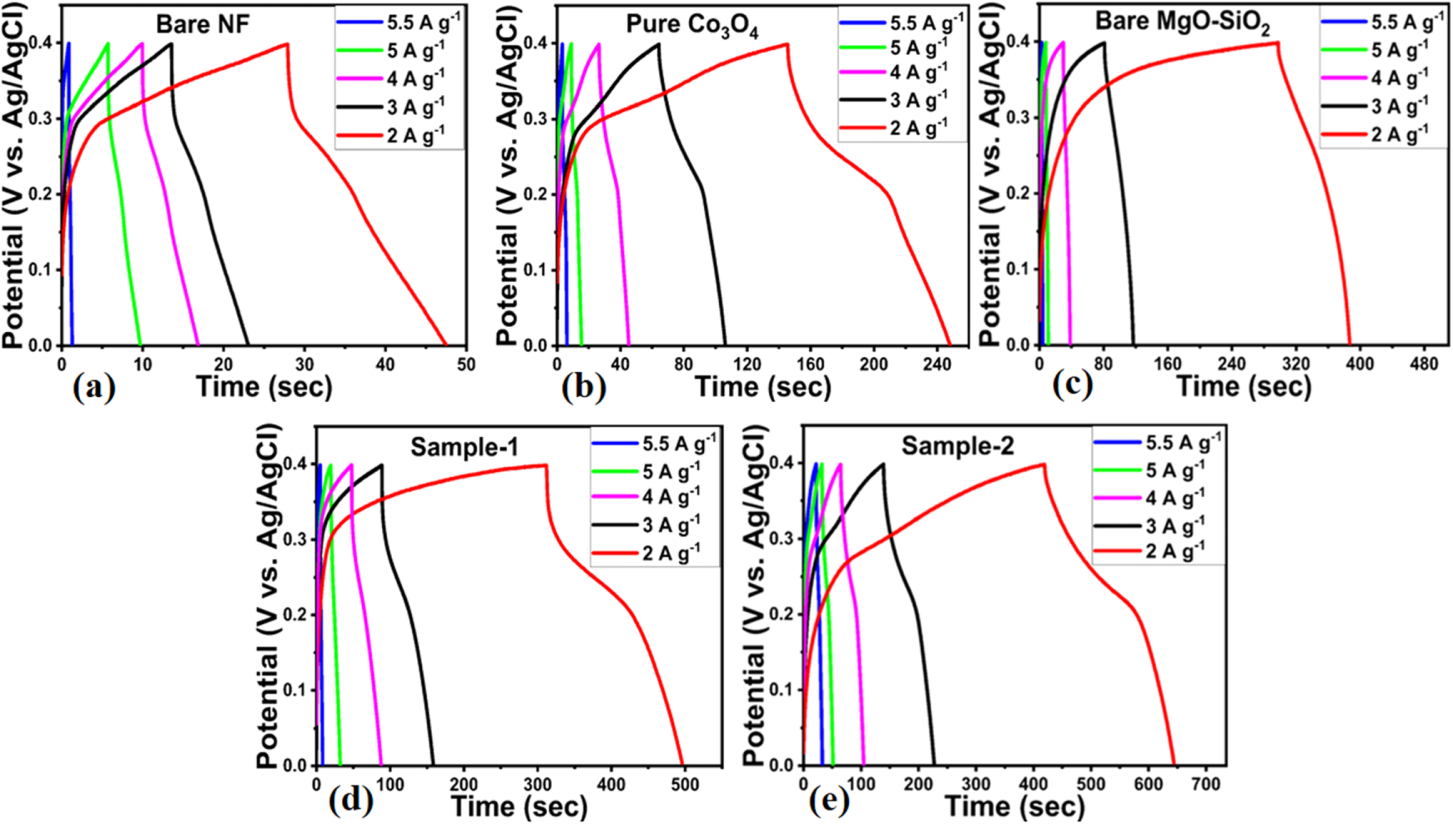
GCD cycles measured at different current densities in 3 M KOH aqueous solution for (a) the bare nickel foam, (b) pure Co_3_O_4_, (c) bare MgO–SiO_2,_ (d) Co_3_O_4_/MgO–SiO_2_ composite (sample 1), and (e) Co_3_O_4_/MgO–SiO_2_ composite (sample 2).

At different current densities, such as 2, 3, 4, 5 and 5.5 A g^−1^, galvanic charge–discharge cycles were measured using a potential window of −0.1 to 5 V for bare nickel foam, pure Co_3_O_4_, bare MgO–SiO_2_ composite, and Co_3_O_4_/MgO–SiO_2_ composites (samples 1 and 2), as shown in Fig. 7a–e.

The composite of Co_3_O_4_/MgO–SiO_2_ (sample 2) demonstrated the longest discharging time and the largest integral area within the GCD cycle among all the electrodes. The charge/discharge performance of the composites was significantly improved owing to the presence of MgO and SiO_2_. Both MgO and SiO_2_ played a vital role towards the enhanced electrochemical performance, as MgO improved the cycling stability and lifetime of the composites,^14^ while SiO_2_ offered unique structural properties for the design of electrode materials.^15^ These composites have modified surface properties with multiple benefits, such as high adsorption of hydroxide ions for fast redox reactions and multichannels for charge transfer; thus, an excellent electrochemical capacitance was observed. Therefore, these aspects of MgO and SiO_2_ in the proposed composites were highly dependent on the content of MgO–SiO_2_ that favored the energy storage performance of active redox material. It was highly consistent with CV behaviour among the electrodes. Therefore, it is important to calculate the specific capacitance of the electrode materials presented using the following equation.^30^

2 *C*_s_ = *I* × *t*/*m* × d*v*

Here, *I* represents the current (A), *t* describes the discharging time (s), *m* is the electrode's loading mass (g), and *v* shows the voltage range (V).

**Fig. 8:**
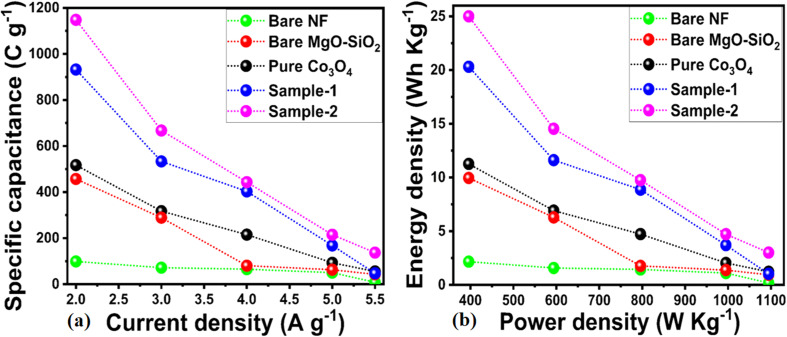
(a) Specific capacitance calculations of the bare nickel foam, pure Co_3_O_4_, bare MgO–SiO_2,_ Co_3_O_4_/MgO–SiO_2_ composite (sample 1), and Co_3_O_4_/MgO–SiO_2_ composite (sample 2) using GCD cycles at different current densities. (b) Energy and power density of specific capacitance calculations of pure Co_3_O_4_, bare MgO–SiO_2,_ Co_3_O_4_/MgO–SiO_2_ composite (sample 1), and Co_3_O_4_/MgO–SiO_2_ composite (sample 2).

The specific capacitances of bare nickel foam, pure Co_3_O_4_, bare MgO–SiO_2_ composite and Co_3_O_4_/MgO–SiO_2_ composites (samples 1 and 2) were calculated using their GCD cycles using eqn (2). Specific capacitances of 98.74, 437, 530, 920, and 1180 F g^−1^ at 2 A g^−1^ were determined for bare nickel foam, pure Co_3_O_4_, bare MgO–SiO_2_ composite, and Co_3_O_4_/MgO–SiO_2_ composites (sample 1 and sample 2), as shown in Fig. 8a. Specific capacitance decreased with increasing current density, and these aspects of specific capacitance were also observed during CV analysis at different scan rates. The CV and GCD analyses demonstrated that the specific capacitance depends on the scan rate and the current density. Sample 2 (Co_3_O_4_/MgO–SiO_2_) exhibited the highest specific capacitance. According to the GCD studies, the nitration time between the electrode material and electrolytic ions is long at low current density ranges, allowing many charges to be stored in the electrode material and resulting in maximum specific capacitance values. The specific capacitance value was low due to the interaction time between the electrode material and electrolytic ions, which was related to a noticeably short interval.^31^ There are two explanations for the large specific capacitance contribution of Co_3_O_4_/MgO–SiO_2_ composite. Firstly, it could be explained by rapid electrochemical reactions such as ion adsorption/desorption and pseudocapacitance followed by faradaic reactions. The second approach involves following the diffusion of ions in the electrolyte solution and electrode material. Thus, both mechanisms play an important role in the electrochemical process and are highly interconnected. The power and energy densities were calculated using eqn (3) and (4).

3 *P* = *E*/Δ*t*

4 *E* = *C*_s_ × Δ*v*^2^/2

here, *p* is power density, *E* is energy, Δ*t* is change in discharge time, *C*_s_ is specific capacitance, and Δ*v* is change in voltage.

The calculations of energy density and power density for bare nickel foam, pure Co_3_O_4_, bare MgO–SiO_2_ composite, and Co_3_O_4_/MgO–SiO_2_ composites (sample 1 and sample 2) are shown in Fig. 8b. It could be seen that the Co_3_O_4_/MgO–SiO_2_ composite (sample 2) exhibited the largest energy density of 25 W h kg^−1^ at a power density of 400 W kg^−1^. A summary of obtained supercapacitor performance based on synthesized materials is shown in Table 1. The electrode performance of Co_3_O_4_/MgO–SiO_2_ (sample 2) was also compared with the existing literature, as shown in ESI Table (S1).† Interestingly, the composite system performs better due to its unique structure and favorable surface for rapid interaction with electrolytic ions.

**Table 1:** Supercapacitor performance evaluation of pure Co_3_O_4_, bare MgO–SiO_2,_ Co_3_O_4_/MgO–SiO_2_ composite (sample 1), and Co_3_O_4_/MgO–SiO_2_ composite (sample 2)

Sample	Current density (A g^−1^)	Specific capacitance (F g^−1^)	Energy density (W h kg^−1^)	Power density (W kg^−1^)
Bare NF	2	98.74	2.16	397.00
	3	71.79	1.57	595.50
	4	65.33	1.44	796.00
	5	50.13	1.11	997.50
	5.5	8.27	0.18	1097.25
Pure Co_3_O_4_	2	517.17	11.26	396.00
	3	317.42	6.91	594.00
	4	215.08	4.73	796.00
	5	92.96	2.05	995.00
	5.5	55.28	1.22	1094.50
Bare MgO–SiO_2_	2	457.07	9.96	396.00
	3	288.64	6.29	594.00
	4	80.40	1.77	796.00
	5	62.81	1.38	995.00
	5.5	42.84	0.94	1094.50
Sample-1	2	932.32	20.31	396.00
	3	533.33	11.62	594.00
	4	403.02	8.87	796.00
	5	168.34	3.70	995.00
	5.5	46.98	1.03	1094.50
Sample-2	2	1147.98	25.00	396.00
	3	667.42	14.54	594.00
	4	443.22	9.75	796.00
	5	214.82	4.73	995.00
	5.5	136.81	3.01	1094.50

### 3.3. Asymmetric supercapacitor device fabrication using the anode of Co_3_O_4_/MgO–SiO_2_ composite (sample 2)

**Fig. 9:**
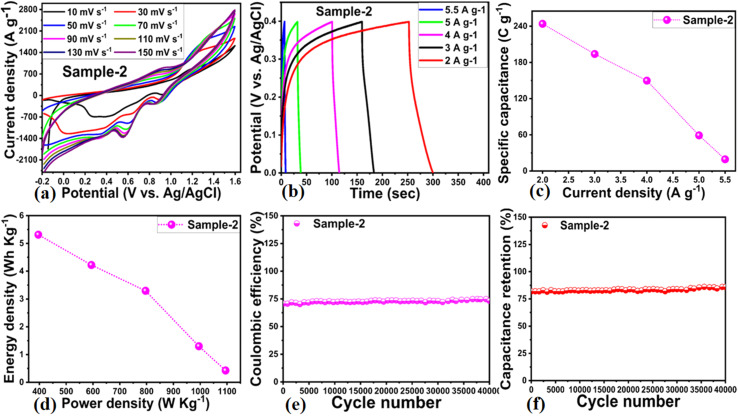
(a) CV cycles at different scan rates of the Co_3_O_4_/MgO–SiO_2_ composite (sample 2) for the demonstration of the asymmetric supercapacitor (ASC). (b) GCD cycles of the Co_3_O_4_/MgO–SiO_2_ composite (sample 2) for the ASC. (c) Specific capacitance calculation of Co_3_O_4_/MgO–SiO_2_ composite (sample 2) using GCD cycles at different current densities. (d) Energy and power density calculation of the Co_3_O_4_/MgO–SiO_2_ composite (sample 2). (e) Coulombic efficiency calculation and (f) capacitive retention % for the illustration of cycling stability in 3 M KOH electrolytic solution.

Considering the practicality of Co_3_O_4_/MgO–SiO_2_ composite (sample 2), an ASC device was developed using a positive electrode of Co_3_O_4_/MgO–SiO_2_ composite (sample 2) and a negative electrode of activated carbon (AC) in 3 M KOH electrolyte. CV of AC was conducted at various scan rates with non-Faradic behavior, as shown in ESI Fig. (S1).† ASC measurements were conducted at different sweeping scan rates with a working voltage range of −0.2 to 1.5 V. As shown in Fig. 9a, the CV measurement of ASC indicates that the device exhibits well-resolved pseudo-capacitance. As the sweeping rate increased, there was no significant change in the CV curve's shape. As shown in Fig. 9b, GCD curves of ASC devices were recorded at different current densities, including 2, 3, 4, 5, and 5.5 A g^−1^. According to Fig. 9c, the specific capacitance of the ASC was 250 F g^−1^ at 2 A g^−1^. The ASC's energy density and power density were also calculated using the GCD curves and the given equations.^32^

5 *P* = *E* × 3600/Δ*t*

6 *E* = *C*_s_ × Δ*v*^2^/7.2

As seen in Fig. 9d, the ASC device offers power and energy densities of 5.5 W h kg^−1^ and 1100 W kg^−1^ at 2 A g^−1^, respectively. As shown in Fig. 9e and f, the cycling stability of the ASC device was evaluated through 40 000 GCD cycles at 2 A g^−1^, along with an estimation of the capacitance retention % and coulombic efficiency of the device. According to Fig. 9e and f, the coulombic efficiency of the ASC was approximately 74.7%, and the capacitance retention percentage was approximately 85.9%. A summary of the ASC performance is given in Table 2.

**Table 2:** Highlights of figures of merit for the ASC device

Sample	Current density (A g^−1^)	Specific capacitance (F g^−1^)	Energy density (W h kg^−1^)	Power density (W kg^−1^)	Coulombic efficiency (%)	Capacitance retention (%)
Sample-2	2	243.94	5.31	396.00	74.7 (40 000 cycles)	85.9 (40 000 cycles)
	3	193.94	4.22	594.00		
	4	149.75	3.29	796.00		
	5	59.05	1.30	995.00		
	5.5	19.35	0.43	1094.50		

### 3.4. Oxygen evolution reaction performance of Co_3_O_4_/MgO–SiO_2_ composites

**Fig. 10:**
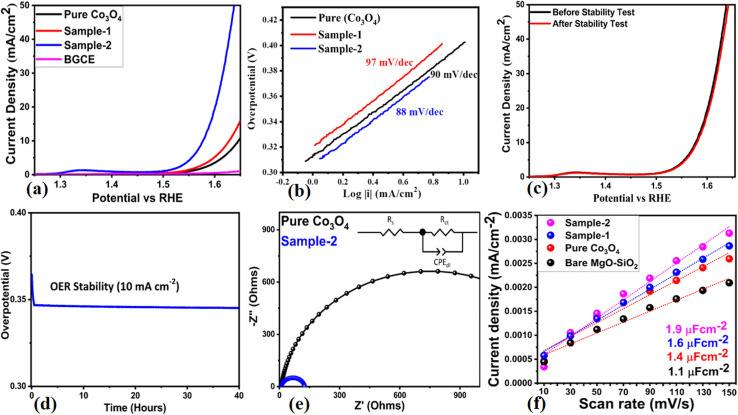
(a) LSV curves of the bare glassy carbon electrode (BGCE), pure Co_3_O_4_, bare MgO–SiO_2,_ Co_3_O_4_/MgO–SiO_2_ composite (sample 1), and Co_3_O_4_/MgO–SiO_2_ composite (sample 2) at 2 mV s^−1^ in 1 M KOH. (b) Corresponding Tafel slope analysis. (c) LSV curves at mV s^−1^ before and after the durability test of the Co_3_O_4_/MgO–SiO_2_ composite (sample 2). (d) Durability study of the Co_3_O_4_/MgO–SiO_2_ composite (sample 2) using chronoamperometry for 40 hours at a fixed current density of 10 mA cm^−2^. (e) Nyquist plots of pure Co_3_O_4_ and Co_3_O_4_/MgO–SiO_2_ composite (sample 2) through EIS measurements. (f) ECSA calculations using non Faradaic CV curves at different scan rates for pure Co_3_O_4_, bare MgO–SiO_2,_ Co_3_O_4_/MgO–SiO_2_ composite (sample 1), and Co_3_O_4_/MgO–SiO_2_ composite (sample 2) .

The electrochemical performances of various materials, including bare glassy carbon electrode (BGCE), pure Co_3_O_4_, bare MgO–SiO_2_ composite, and Co_3_O_4_/MgO–SiO_2_ composites (samples 1 and 2), were also investigated for electrochemical water splitting, especially the oxygen evolution reaction (OER) in 1 M KOH electrolytic solution. A slow scan CV at 5 mV s^−1^ stabilized the electrode, followed by linear sweep voltammetry (LSV) at a scan rate of 2 mV s^−1^. In Fig. 10a, it is evident that the OER activity of Co_3_O_4_/MgO–SiO_2_ composite (sample 2) was excellent, indicating the dual role of electrode materials for renewable energy generation and storage. At 10 mA cm^−2^, the overpotentials for pure Co_3_O_4_, Co_3_O_4_/MgO–SiO_2_ composite (sample 1) and Co_3_O_4_/MgO–SiO_2_ composite (sample 2) were found to be 410 mV, 390 mV, and 340 mV, respectively. The OER activity of BGCE was negligible, as shown in Fig. 10. Co_3_O_4_/MgO–SiO_2_ composite (sample 2) exhibits enhanced electrochemical activity due to its enriched surface-active sites, rapid charge transport, and high compatibility with electrode surfaces. In addition, the OER kinetics were examined through the linear portion of the LSV by applying the Tafel equation, and the corresponding Tafel values are shown in Fig. 10b. Based on the Tafel slopes of pure Co_3_O_4_ and Co_3_O_4_/MgO–SiO_2_ composites (samples 1 and 2), values of 97 mV dec^−1^, 90 mV dec^−1^, and 88 mV dec^−1^, respectively, were obtained. Tafel analysis demonstrated that the Co_3_O_4_/MgO–SiO_2_ composite (sample 2) exhibits favorable OER kinetics at significantly high rates. Fig. 10c illustrates the stability of the Co_3_O_4_/MgO–SiO_2_ composite (sample 2) before and after the durability test for 40 hours. There was a high degree of overlap between the LSV curves without any variation in the onset potential or overpotential, so MgO–SiO_2_ composite induction into Co_3_O_4_ greatly improved electrode stability. As seen in Fig. 10d, chronopotentiometry was used to determine the variation of the OER overpotential for 40 hours at a fixed current density of 10 mA cm^−2^. The Co_3_O_4_/MgO–SiO_2_ composite (sample 2) demonstrated excellent durability for 40 hours without any fluctuation in the overpotential. As shown in Fig. 10e, Nyquist plots for pure Co_3_O_4_ and Co_3_O_4_/MgO–SiO_2_ composite (sample 2) were measured using EIS measurements with the experimental conditions of 100 kHz to 0.1 Hz, 5 mV amplitude, and the OER onset potential. Using Z-View software, raw EIS data were simulated and well-fit equivalent circuits were obtained containing circuit elements such as solution resistance (*R*_s_), charge transfer resistance (*R*_ct_), and capacitance double layer (*C*_dl_). Nyquist plots of pure Co_3_O_4_ showed arc areas indicating significant resistance; however, Nyquist plots of Co_3_O_4_/MgO–SiO_2_ composite (sample 2) showed small arcs indicating rapid charge transfer rates between electrodes and electrolytes. The induction of the MgO–SiO_2_ composite into Co_3_O_4_ significantly enhanced the charge transport of the composite, potentially favoring electrochemical reactions according to the EIS analysis.

**Fig. 11:**
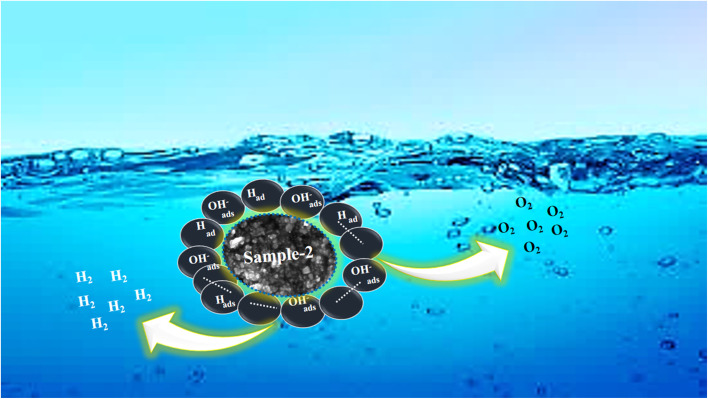
Illustration of the generalized OER mechanism under alkaline conditions.

Electrochemical active surface area (ECSA) was used to investigate the electrocatalytic properties of pure Co_3_O_4_, bare MgO–SiO_2_ and Co_3_O_4_/MgO–SiO_2_ composites (sample 1 and sample 2).^33–49^ The following mathematical relationship was used to estimate the ECSA.ECSA = *C*_dl_/*C*_s_6Here, *C*_dl_ is double-layer capacitance and *C*_s_ is the specific capacitance of 0.040 mF cm^−2^ in 1 M electrolytic solution of KOH. The ECSA was obtained using CV cycles at various scan rates from 10 mV s^−1^ to 150 mV s^−1^, as shown in ESI Fig. (S2).† The calculation of capacitive currents (Δ*j* = *j*_a_ − *j*_c_) is based on the sweeping rate (*v*). Here, the linear fitting slope was considered twice that of the capacitance double layer, as shown in Fig. 10f. The observed *C*_dl_ values for the pure Co_3_O_4_, bare MgO–SiO_2_ and Co_3_O_4_/MgO–SiO_2_ composites (sample 1 and sample 2) were 1.4, 1.1, 1.6 and 1.9 μF cm^−2^, respectively. The ECAS value will be twice that of the *C*_dl_ of each material. Overall, the electrochemical performance of Co_3_O_4_/MgO–SiO_2_ (sample 2) could be attributed to the enhanced surface area, abundant active sites, rapid charge transfer rate, large interaction with electrolytic ions and high compatibility with the surface of the electrode. The comparative study of the OER performance of Co_3_O_4_/MgO–SiO_2_ (sample 2) is given in ESI Table (S2).† The performance of the presented Co_3_O_4_/MgO–SiO_2_ (sample 2) is equal or superior to those of many of the recent electrocatalysts in terms of synthesis, OER activity, and low cost. The generalized OER mechanism on the proposed electrocatalytic material (sample 2) in the alkaline electrolyte is shown in Fig. 11.

## 4. Conclusions

For the first time, we report the synthesis of Co_3_O_4_/MgO–SiO_2_ composites using orange peel extract during hydrothermal treatment. In crystalline form, the composites displayed compact nanoparticles and cubic phases of Co_3_O_4_ and MgO. As a result of electrochemical studies, the Co_3_O_4_/MgO–SiO_2_ composite (sample 2) showed excellent energy storage characteristics when used to develop an ASC device, with a specific capacitance of 243 F g^−1^ at 2 A g^−1^ as well as excellent cycling stability for 40 000 GCD cycles with a retention of capacitance of 85.7%. An overpotential of 340 mV at 10 mA cm^−2^ was also observed for this composite (sample 2). The composite made from Co_3_O_4_/MgO–SiO_2_ (sample 2) proved to be exceptionally durable after 40 hours of testing. As a result of the induction of MgO–SiO_2_ composite into Co_3_O_4_, the charge transfer rate, surface area, and compatibility with the electrode surface were enhanced, resulting in excellent electrochemical performance.

## Data availability

The data are available from the corresponding author upon request.

## Author's contribution

Gulzar Ali: material synthesis and partial electrochemical tests. Aneela Tahira: XRD analysis and wrote the draft. Asma Hayat: SEM analysis and wrote the draft. Muhammad Ali Bhatti: partial electrochemical tests. Aqeel Ahmed Shah: SEM measurements. Syed Nizam Uddin Shah Bukhari: optical band gap analysis. Elmuez Dawi: review and editing of the original draft of the manuscript. Ayman Nafady: OER analysis and involved in the write up. Riyadh H. Alshammari: supercapacitor analysis. Matteo Tonezzer: FTIR analysis and wrote the draft. Muhammad Kashif Samoon: EDS mapping. Zafar Hussain Ibupoto: main supervision, and writing of the original draft of manuscript.

## Conflicts of interest

Authors have no conflict of interest in the presented research work.

## Supplementary Material

RA-014-D4RA07337A-s001

## References

[cit1] Xie L. J., Wu J. F., Chen C. M., Zhang C. M., Wan L., Wang J. L., Kong Q. Q., Lv C. X., Li K. X., Sun G. H. (2013). J. Power Sources.

[cit2] Sephra P. J., Baraneedharan P., Sivakumar M., Thangadurai T. D., Nehru K. (2018). Mater. Res. Bull..

[cit3] Simon P., Gogotsi Y., Dunn B. (2014). Science.

[cit4] Zhang Q., Uchaker E., Candelaria S. L., Cao G. (2013). Chem. Soc. Rev..

[cit5] Chen Z., Zhao W., Liu Q., Xu Y., Wang Q., Lin J., Wu H. B. (2024). Nano-Micro Lett..

[cit6] Jow T. R., Zheng J. P. (1995). Mater. Res. Soc. Symp. Proc..

[cit7] Lokhande C. D., Dubal D. P., Joo O. S. (2011). Curr. Appl. Phys..

[cit8] Farhadi S., Safabakhsh J., Zaringhadam P. (2013). J. Nanostructure Chem..

[cit9] Cheng Y., Wang Y., Chen B., Han X., He F., He C., Hu W., Zhou G., Zhao N. (2024). Adv. Mater..

[cit10] Iro Z. S., Subramani C., Kesavan T., Dash S. S., Sasidharan M., Sundramoorthy A. K. (2017). Mater. Res. Express.

[cit11] Sravani B., Maseed H., Chandrasekhar Y., Srikanth V. V. S. S., Madhavi G. (2019). Nanoscale.

[cit12] Bajdich M., Nørskov J. K., Vojvodic A. (2015). Phys. Rev. B:Condens. Matter.

[cit13] He X., Tan J., Wei J., Yin F., Chen B., Liang X., Li G. (2020). Appl. Surf. Sci..

[cit14] Kweon H. J., Kim S. J., Park D. G. (2000). J. Power Sources.

[cit15] Iro Z. S., Subramani C., Kesavan T., Dash S. S., Sasidharan M., Sundramoorthy A. K. (2017). Mater. Res. Express.

[cit16] Prestat M. (2023). J. Power Sources.

[cit17] Kusoglu A. (2021). Electrochem. Soc. Interface.

[cit18] Bukhari S. N. U. S., Shah A. A., Bhatti M. A., Tahira A., Channa I. A., Shah A. K., Chandio A. D., Mahdi W. A., Alshehri S., Ibhupoto Z. H., Liu W. (2022). Nanomaterials.

[cit19] She Z. W., Kibsgaard J., Dickens C. F., Chorkendorff I., Nørskov J. K., Jaramill T. F. (2017). Science.

[cit20] Suen N. T., Hung S. F., Quan Q., Zhang N., Xu Y. J., Chen H. M. (2017). Chem. Soc. Rev..

[cit21] Bukhari S. N. U. S., Shah A. A., Liu W., Channa I. A., Chandio A. D., Chandio I. A., Ibupoto Z. H. (2024). Ceram. Int..

[cit22] Dyar M. D., Wiedenbeck M., Robertson D., Cross L. R., Delaney J. S., Ferguson K., Francis C. A., Grew E. S., Guidotti C. V., Hervig R. L., Hughes J. M. (2021). Geostand. Newsl..

[cit23] Ni S., Chou L., Chang J. (2007). Ceram. Int..

[cit24] Alrehaily L. M., Joseph J. M., Biesinger M. C., Guzonas D. A., Wren J. C. (2013). Phys. Chem. Chem. Phys..

[cit25] Shah A. A., Bhatti M. A., Tahira A., Chandio A. D., Channa I. A., Sahito A. G., Chalangar E., Willander M., Nur O., Ibupoto Z. H. (2020). Ceram. Int..

[cit26] Bhargava R., Khan S., Ahmad N., Ansari M. M. N. (2018). AIP Conf. Proc..

[cit27] Dubey S., Kumar J., Kumar A., Sharma Y. C. (2018). Adv. Powder Technol..

[cit28] Najib S., Erdem E. (2019). Nanoscale Adv..

[cit29] Ali F., Khalid N. R., Nabi G., Ul-Hamid A., Ikram M. (2021). Int. J. Energy Res..

[cit30] Shaheen I., Ahmad K. S., Zequine C., Gupta R. K., Thomas A. G., Malik M. A. (2021). Energy.

[cit31] Sankar K. V., Selvan R. K., Meyrick D. (2015). RSC Adv..

[cit32] Zhang L., Zheng W., Jiu H., Ni C., Chang J., Qi G. (2016). Electrochim. Acta.

[cit33] Martínez-Hincapié R., Wegner J., Anwar M. U., Raza-Khan A., Franzka S., Kleszczynski S., Čolić V. (2024). Electrochim. Acta.

[cit34] Pathak M., Jose J. R., Chakraborty B., Rout C. S. (2020). J. Chem. Phys..

[cit35] Wang H., Gao Q., Jiang L. (2011). Small.

[cit36] Kumar S., Tahira A., Bhatti A. L., Bhatti M. A., Mari R. H., Shaikh N. M., Ibupoto Z. H. (2023). RSC Adv..

[cit37] Kumar S., Tahira A., Emo M., Vigolo B., Infantes-Molin A., Alotaibi A. M., Ibupoto Z. H. (2023). J. Energy Storage.

[cit38] Kumar S., Tahira A., Bhatti A. L., Bhatti M. A., Ujjan Z. A., Aftab U., Kumar S., Al-Kahtani A. A., Nafady A., Dawi E., Emo M. (2024). J. Energy Storage.

[cit39] Hayat A., Tahira A., Bhatti M. A., Naz I., Shah A. A., Dawi E., Tonezzer M., Nafady A., Alshammari R. H., Ibupoto Z. H. (2024). Results Eng..

[cit40] Bhatti A. L., Tahira A., Kumar S., Ujjan Z. A., Bhatti M. A., Kumar S., Aftab U., Karsy A., Nafady A., Infantes-Molina A., Ibupoto Z. H. (2023). RSC Adv..

[cit41] Maseed H., Srikanth V. V., Narayana A. L., Hussain O. M., Shaikshavali P. (2020). Mater. Lett..

[cit42] Kurtan U., Aydın H., Büyük B., Şahintürk U., Almessiere M. A., Baykal A. (2020). J. Energy Storage.

[cit43] Rotte N. K., Naresh V., Muduli S., Reddy V., Srikanth V. V. S., Martha S. K. (2020). Electrochim. Acta.

[cit44] Yang L., Zhang L., Xu G., Ma X., Wang W., Song H., Jia D. (2018). ACS Sustain. Chem. Eng..

[cit45] Wang Y., Zhang Z., Liu X., Ding F., Zou P., Wang X., Rao H. (2018). ACS Sustain. Chem. Eng..

[cit46] Mahala C., Basu M. (2017). ACS Omega.

[cit47] Li Z., Li B., Chen J., Pang Q., Shen P. (2019). Int. J. Hydrogen Energy.

[cit48] Li Y., Hu L., Zheng W., Peng X., Liu M., Chu P. K., Lee L. Y. S. (2018). Nano Energy.

[cit49] Zhang L., Yuan H., Li X., Wang Y. (2022). Coatings.

