# Supplementary material for: Facile and ecofriendly green synthesis of Co3O4/MgO–SiO2 composites towards efficient asymmetric supercapacitor and oxygen evolution reaction applications
Source: RSC Adv. 2024 Nov 28;14(51):38009–21. doi: 10.1039/d4ra07337a (PMC11604033; doi:10.1039/d4ra07337a)
Supplement: RA-014-D4RA07337A-s001 [file RA-014-D4RA07337A-s001.pdf]

Supplementary Information (SI) for RSC Advances.
This journal is © The Royal Society of Chemistry 2024

Supplementary Information

Facile and ecofriendly green Synthesis of Co_3O_4/MgO-SiO_2 Composites towards efficient asymmetric supercapacitor and oxygen evolution reaction applications

Gulzar Ali[1], Aneela Tahira[2], Asma Hayat[1], Muhammad Ali Bhatti[3], Aqeel Ahmed Shah[4], Syed Nizam Uddin Shah Bukhari[5], Elmuez Dawi[6], Ayman Nafady[7], Riyadh H. Alshammari[7], Matteo Tonezzer[8], Muhammad Kashif Samoon[9], Zafar Hussain Ibupoto[*1]

[1]Institute of Chemistry, University of Sindh, Jamshoro 76080, Pakistan.
gulzaralichemist@gmail.com ; asmabaloch141617@gmail.com ; zaffar.ibhupoto@usindh.edu.pk

[2]Institute of Chemistry, Shah Abdul Latif University Khairpur Mirs, Sindh, Pakistan.
aneela.tahira@salu.edu.pk

[3]Centre for Environmental Sciences, University of Sindh Jamshoro, 76080, Sindh, Pakistan.
mali.bhatti@usindh.edu.pk

[4]Wet Chemistry Laboratory, Department of Metallurgical Engineering, NED University of Engineering and Technology, University Road, Karachi 75270, Pakistan.
aqeelshah@cloud.neduet.edu.pk

[5]Department of Basic Science and Humanities, Dawood University of Engineering and Technology, Karachi, Sindh, 74800 Pakistan. nizamuddin@duet.edu.pk

[6]College of Humanities and Sciences, Department of Mathematics and Sciences, Ajman University, P.O. Box 346, Ajman, UAE. e.dawi@ajman.ac.ae

[7]Chemistry Department, College of Science, King Saud University, Riyadh, 11451, Saudi Arabia. anafady@ksu.edu.sa ; ralshammari@ksu.edu.sa

[8]Department of Chemical and Geological Sciences, University of Cagliari, Monserrato, Italy.
matteo.tonezzer@cnr.it

[9]Centre for Pure and Applied Geology, University of Sindh Jamshoro, Jamshoro, Sindh, 76080, Pakistan. kashif.samoon@usindh.edu.pk

[*]Corresponding author(s):
Zafar Hussain Ibupoto, PhD, Email: zaffar.ibhupoto@usindh.edu.pk

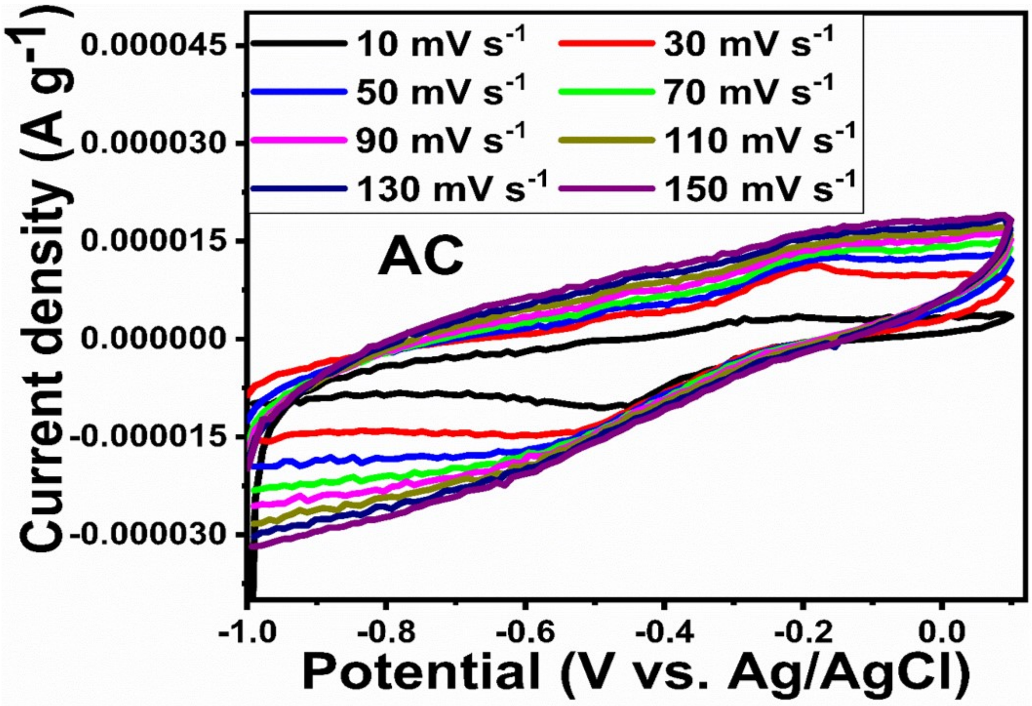

Figure (S1): CV curves of rice husk derived activated carbon (Ac) at various scan rate s in 1M KOH aqueous solution.

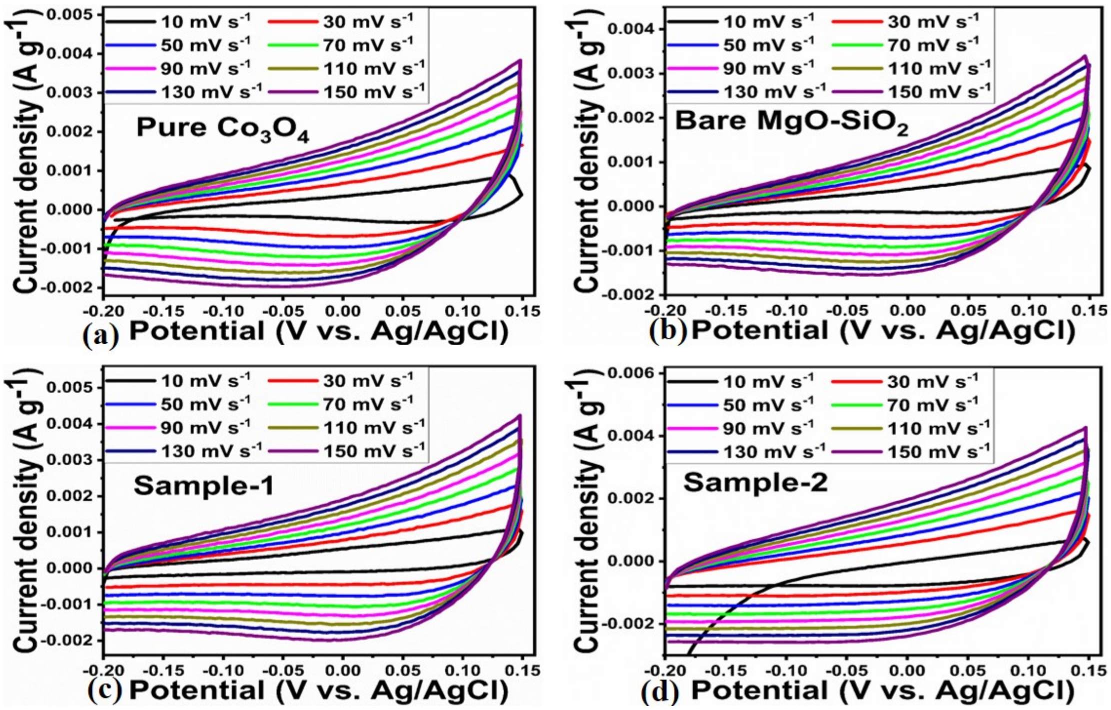

Figure (S2): CV curves at various scan rates in 1M KOH aqueous solution (a) pure Co_3O_4, (b) bare MgO-SiO_2, (c) Co_3O_4/MgO-SiO_2 composite (sample1), (d) Co_3O_4/MgO-SiO_2 composite (sample 2).

Table (S1): Comparative analysis of presented supercapacitor based on Co_3O_4/MgO-SiO_2 composite (sample 2).

**Material**	**Specific Capacitance (F/g)**	**Current Density (A/g)**	**Potential Window (V)**	**Energy Density (Wh kg^{-1})**	**Power Density (W kg^{-1})**	**Reference**
MWCNTs	84 F/g	0.6 A/g	-0.5 to 2.2 V	21	6237	[35]
Ni–Co–O–1	568 F/g	20 A/g	0 to 0.5 V	19.72	5000	[36]
$NiCo_2O_4$ (Lemon juice)	358 F/g	0.8 A/g	0 to 0.4 V	7.96	160	[37]
$NiCo_2O_4$ (Grapefruit)	434 F/g	0.8 A/g	0 to 0.4 V	9.64	160	[38]
$NiCo_2O_4$ (OP-1)	997 F/g	1.5 A/g	0 to 0.4 V	22.17	300	[39]

Sample 1 Co_3O_4	867 F g^{-1}	1.5 Ag^{-1}	0 to 0.4 V	18.41	239.25	[40]
Co_3O_4	699 F g^{-1}	0.8 A g^{-1}	0 to 0.4 V	30	236	[41]
MnO/FLG	778.5	0.5 A g^{-1}	0 to 1 V	81	292	[42]
CoNi-alloy	132 Fg^{-1}	1 Ag^{-1}	0 to 1 V	4.60	250	[43]
S, N-FLG	298 F g^{-1}	1 A g^{-1}	0 to -0.6	15	298	[44]
**Sample-2**	**1147.98**	**2 A/g**	**0 to 0.4 V**	**25.00**	**396.00**	**This Work**

Table (S2(: OER comparative study of Co_3O_4/MgO-SiO_2 composite (sample 2).

Catalyst	Over potential (mV) @ 10 mA/cm^2	Electrolyte	References
CoS_x/MoS_2	347	1.0 M KOH	[45]
MOF derived-$NiCo_2O_4$ / NiO	430	1.0 M KOH	[46]
$NiCo_2O_4$/NiO	360	1.0 M KOH	[47]
$NiCo_2O_4$@Graphene nanosheets	383	1.0 M KOH	[48]
$NiCo_2O_4$@$NiCo_2O_4$	440	1.0 M KOH	[49]
NiCo film	422	0.5 M KOH	[50]
**Sample-2**	**340**	**1.0 M KOH**	**This Work**
